# Associations between early term and late/post term infants and development of epilepsy: A cohort study

**DOI:** 10.1371/journal.pone.0210181

**Published:** 2018-12-31

**Authors:** David Odd, Alessandra Glover Williams, Cathy Winter, Timothy Draycott

**Affiliations:** 1 Department of Women’s and Children’s Health, North Bristol NHS Trust, Bristol, United Kingdom; 2 Population Health Sciences, Bristol Medical School, University of Bristol, Bristol, United Kingdom; 3 Royal United Hospital, Bath, United Kingdom; TNO, NETHERLANDS

## Abstract

**Background:**

While life-long impacts exist for infants born one or two weeks early little evidence exists for those infants born after their due date. However interventions could be used to expedite birth if the risks of continuing the pregnancy are higher than intervening. It is known that the risk of epilepsy in childhood is higher in infants exposed to perinatal compromise and therefore may be useful as a proxy for intrapartum compromise. The aim of this work is to quantify the likelihood of children developing epilepsy based on their gestational age at birth (37–39 weeks or ≥41 weeks).

**Methods:**

The work is based on term infants born in Sweden between 1983 and 1993 (n = 1,030,168), linked to data on disability pension, child mortality and in-patient epilepsy care. The reference group was defined as infants born at 39 or 40 completed weeks of gestation; compared with infants born at early term (37/38 weeks) or late/post term (41 weeks or more). Primary outcome was defined a-priori as a diagnosis of epilepsy before 20 years of age. Secondary outcomes were childhood mortality (before five years of age), and registered for disability pension before 20 years of age. Logistic regression models were used to assess any association of the outcomes with gestational age at birth.

**Findings:**

In the unadjusted results, infants born 7 or more days after their due date had higher risks of epilepsy and disability pension than the reference group, but similar risks of child death. Early term infants showed higher risks of epilepsy, disability pension and child death. After adjustment for confounders, there remained a higher risk of epilepsy for both early term (OR 1·19 (1·11–1·29)) and late/post term infants (OR 1·13 (1·06–1·22)).

**Interpretation:**

Infants born at 37/38 week or 41 weeks and above, when compared to those born at 39 or 40 weeks gestation, have an increased risk of developing epilepsy. This data could be useful in helping women and care givers make decisions with regard to the timing of induction of labour.

## Introduction

While pregnant mothers report that the health of their unborn infant is the single biggest priority during labor and birth[1] the optimal time management of pregnancies that approach their due date is debated[2] although recent work suggests that earlier induction is associated with a reduction in cesarean birth with possible improvements for infant outcomes[3]. Although prediction of which infants will become compromised around birth is poorly understood[4], we have presented some work that shows that modelling of risk is feasible[5] and that simple interventions can improve neonatal and maternal outcomes[6].

While most infants are born at 39 or 40 weeks gestation; many are born shortly before or after. There is now evidence that infants born at 37 and 38 weeks (Early Term) are at higher risk of neurodevelopmental problems[7,8] but less evidence exists for those born after their due date[9,10]. Infants who remain *in-utero* after their due date are exposed to increasing risk of infection, late stillbirth and increased risks of complications, such as shoulder dystocia and perinatal asphyxia[11], without obvious benefits to the infant. The current literature on late or post term outcomes is based on short term observations and, like the effect of preterm birth, the neurological impacts may be substantially greater in the long term[12], even for infants without signs in the newborn period. In contrast to premature birth, delivery of infants beyond their due date may reduce risk, for either the mother or the infant if an adverse outcome looked likely. Induction of labour (IOL), appears to be beneficial in certain high risk groups[13–16] but its place in late or post term management not yet clear[16,17]. The UK National Institute for Health and Care Excellence (NICE) suggest a key research priority is to "identify babies at particularly high risk of morbidity and mortality who will benefit from induction and therefore avoid induction for babies who do not need it"[18], while the prediction of which infants will become compromised around birth has been identified as a priority by the RCOG[19] and the UK Department of Health[20].

One important neurological condition, known to be associated with fetal compromise and perinatal asphyxia is the development of later epilepsy[21] and risk may increase in a dose-response pattern after even mild degrees of compromise at birth[22] without obvious brain injury after birth. As such it may be represent a pragmatic outcome of perinatal brain injury even in infants without evidence of widespread impairment after birth, and we hypothesized *a-priori* that rates may be higher in infants born over one week early or late compared to those infants born within 7 days of their due date.

The primary aim of this work was to (i) quantify the risks of birth at 37/38 week gestation and at, or after 41 completed weeks of gestation, on the risk of later development of epilepsy, with particular emphasis on those born late or post term (>40 weeks); and (ii) to investigate if specific sub-groups appear at significantly higher risk, that then may benefit from enhanced surveillance or earlier birth.

## Materials and methods

The initial dataset was based on the Swedish Birth Register and contained information on 2,650,219 eligible infants born in Sweden between 1973–2012, at 37 weeks or more of gestation without congenital abnormalities. Linkage to other datasets (see below) was only possible when the mother was born between 1948 and 1973 (inclusive). In order to develop a population-based cohort, with 20 years of follow-up data for the infants the main cohort is restricted to infants born between 1983 and 1993, by mothers aged between 20 and 35 years of age at the time of delivery (n = 1,049,497). For the main analyses, infants with missing data on *a priori* confounders (n = 19,329 (1.2%)) were removed; leaving 1,030,168 children for the main analyses (S1 Fig).

Perinatal data was derived from the medical birth registry which provides data on 98% to 99% of births in Sweden and includes Apgar scores, as well as both neonatal and maternal diagnoses (coded using the International Classification of Diseases, 8th, 9th and 10th revisions)[23]. The method of gestational age assessment is not recorded. By using the Swedish unique ID numbers record linkages were made to the Longitudinal Integration Database for Health Insurance and Labour Market Studies (LISA) database (1990–2012) for educational measures and occupational socio-economic data, the Register of the Social Insurance Office to obtain data on diagnosis at attainment of disability pension (1980–2013) and the Inpatient Care Register (1973–2013) for diagnosis of epilepsy (ICD codes 345, G40, G41). Personal ID numbers were deleted before delivery to one of the collaborators (FR). Due to using anonymous registry data, informed consent was neither required nor possible to obtain. Ethical approval was obtained from the Ethical Review Board of Stockholm (Ref: 2015/1279-31/2).

In view of the possible increased risks at both early term (37–38 weeks) and late or post term (41+ weeks), the reference groups were defined as infants born at full term (39 or 40 completed weeks of gestation)[24]; and compared to infants born earlier or later.

The primary outcome was diagnosis of epilepsy before 20 years of age (derived from the inpatient register) after birth seven, or more, days after the infants’ due date (the late or post term group). Secondary outcomes were childhood mortality (before five years of age), and registered for disability pension before 20 years of age. The association with early term birth was also derived.

Covariates were selected as presumed confounders *a-priori*, and categorised into three groups: i) social (maternal age, maternal occupation, maternal education), ii) antenatal (infant sex and primiparity), and ii) intrapartum and neonatal factors (maternal or infant infection, birth weight (z-score), birth by caesarean section).

### Statistical analysis

Initially, subjects with and without missing data were compared. The distribution of risk factors and potential confounders was investigated, according to their gestational age category at birth (i.e. early term, full term (reference) and late or post term). In univariable analysis the risk of epilepsy and the secondary outcomes was derived, split by each completed week of gestation.

In the multivariable analyses, logistic regression models were used to assess any association of the outcomes with gestational age at birth. Random effects models (using birth year and maternal ID as the grouping variables) were used to correct for any trends in practice or diagnosis over the study period as per our previous work[25]. Adjustment for confounders was performed by adding the variables described above to the models in blocks of common variables (social, antenatal, and then intrapartum and neonatal factors). Birthweight and maternal age were assumed to be continuous variable and not grouped further beyond their initial units (grams and years respectively).

To investigate if specific sub-groups appear at significantly higher risk, the model was repeated assessing if possible risk factors modified the association between gestational age at birth and the development of epilepsy. Models had the risk actors added as interactive terms and models were compared using the likelihood ratio test. Factors assessed were; maternal age (above or below 30 years of age), low birth weight (>2 standard deviations (SD) below the gestational mean), multiple birth, sex, parity, caesarean section, and pre-eclampsia.

A number of sensitivity analyses were performed (using the same primary outcome):

1. To test if the risk of epilepsy increased or decreased as the pregnancy progressed beyond the due date, the model was repeated using the number of weeks of birth the infant was born after their due date as an ordinal variable.
2. Splitting the late and post term infants to give 4 exposure groups (37/38 weeks, 39/40 weeks (Ref.), 41 weeks and 42 of more weeks of gestation).
3. We repeated the final analysis using the main analysis cohort (n = 1,030,168); but excluding those infants with evidence of perinatal encephalopathy after birth (n = 356) or those born at 45 weeks gestation (n = 776)
4. We repeated the final analysis based on all infants recorded in the Medical Birth Register at 37 weeks or more of gestation, without congenital abnormalities, born in Sweden between 1973–2012, and with data on exposure, confounders and outcome (epilepsy), irrespective of the length of follow-up data available (n = 2,516,997).
5. The final analysis was repeated twice, using development of epilepsy between the age of 0 and 9 years of age, and between 10 and 19 years of age, when compared to full term infants.

All analyses were conducted with Stata 14 software (Stata Corp, College Station, TX), and results are presented as number (percent), mean (SD), geometric mean (95% confidence interval), or OR (95% confidence interval).

### Role of the funding source

The funder had no role in the study design, data collection, analysis, interpretation of the data, writing of the report or decision to submit the paper for publication.

## Results

A total of 1,030,168 infants were included in the main analyses, while 19,329 eligible infants had insufficient data for inclusion. Infants excluded due to missing data differed from those included in a number of ways (S1 Table). There was little evidence that infants with missing data had different gestational ages or higher risks of epilepsy but they weighed less and had younger, primiparous mothers. Maternal infection appeared less commonly in infants excluded and both maternal occupation and educational status demonstrated a different profile. Infants excluded appeared to have similar one minute Apgar scores, but slightly higher five minute score, and similar risks of encephalopathy.

In total, 263,999 (25·6%) infants were born at 41 weeks gestation or later, while 207,633 (20·2%) were born at 37 or 38 weeks (Table 1). Most demographics varied significantly by gestational age group although absolute differences were small. The highest risk of pre-eclampsia or birth by caesarean section did however appear to be in those infants born at 39 or 40 weeks gestation, while primparous women appeared to be more likely to have late or post term infants than the other two groups. In addition infants born earlier or later than the reference group had lower Apgar scores and a higher risk of encephalopathy consistent with our previous work[25]. A more detailed comparison is shown in S2 Table.

**Table 1:** Characteristics of the study population according to gestational age (n = 1,030,168).

Measure	39 or 40 weeks (n = 558,536) (Reference Group)	37 or 38 weeks (n = 207,633)	≥41 weeks (n = 263,999)	p
**Antenatal Factors**				
Male	281483 (50·4%)	106,781 (51·4%)	139,454 (52·8%)	<0·0001
Birthweight (g)	3581 (451)	3253 (462)	3801 (469)	<0·0001
Pre-eclampsia	8,562 (1·5%)	4982 (2·4%)	3487 (1·3%)	<0·0001
**Intrapartum Factors**				
Maternal Infection	2318 (0·4%)	1160 (0·6%)	1109 (0·4%)	<0·0001
Neonatal Infection	1028 (0·2%)	471 (0·2%)	705 (0·3%)	<0·0001
Caesarean Section	32,960 (5·9%)	45,838 (22·1%)	19,467 (7·4%)	<0·0001
**Demographic factors**				
Maternal Age	28·4 (4·5)	28·6 (4·6)	28·5 (4·5)	<0·0001
Primiparae	218,739 (39·2%)	78,719 (37·9%)	116,045 (44·0%)	<0·0001
Maternal Occupation				<0·0001
Manual	58,343 (10·5%)	22,703 (10·9%)	26,861 (10·2%)	
Non-manual	177,222 (31·7%)	68,521 (33·0%)	80,423 (30·5%)	
Other	322,971 (57·8%)	116,409 (56·1%)	156,715 (59·4%)	
Maternal Education				<0·0001
<9 Years	61,381 (11·0%)	26,234 (12·6%)	27,432 (10·4%)	
9–10 Years	286,739 (51·3%)	107,704 (51.9%)	133,040 (50.4%)	
Full Secondary	206,238 (36·9%)	72,269 (34·8%)	101,451 (38·4%)	
Higher Education	4,178 (0·8%)	1,426 (0·7%)	2,076 (0·8%)	
**Birth Characteristics**				
Apgar Score				
1 minute	8·75 (8·75–9·76)	8·73 (8·73–8·74)	8·59 (8·58–8·59)	0·0001
5 minute	9·77 (9·77–9·77)	9·73 (9·72–9·73)	9·70 (9·70–9·70)	0·0001
Encephalopathy	161 (0·03%)	80 (0·04%)	115 (0·04%)	0·0020

Values are number (%), mean (±SD) or geometric mean (95% confidence interval) as appropriate·

**Fig 1:**
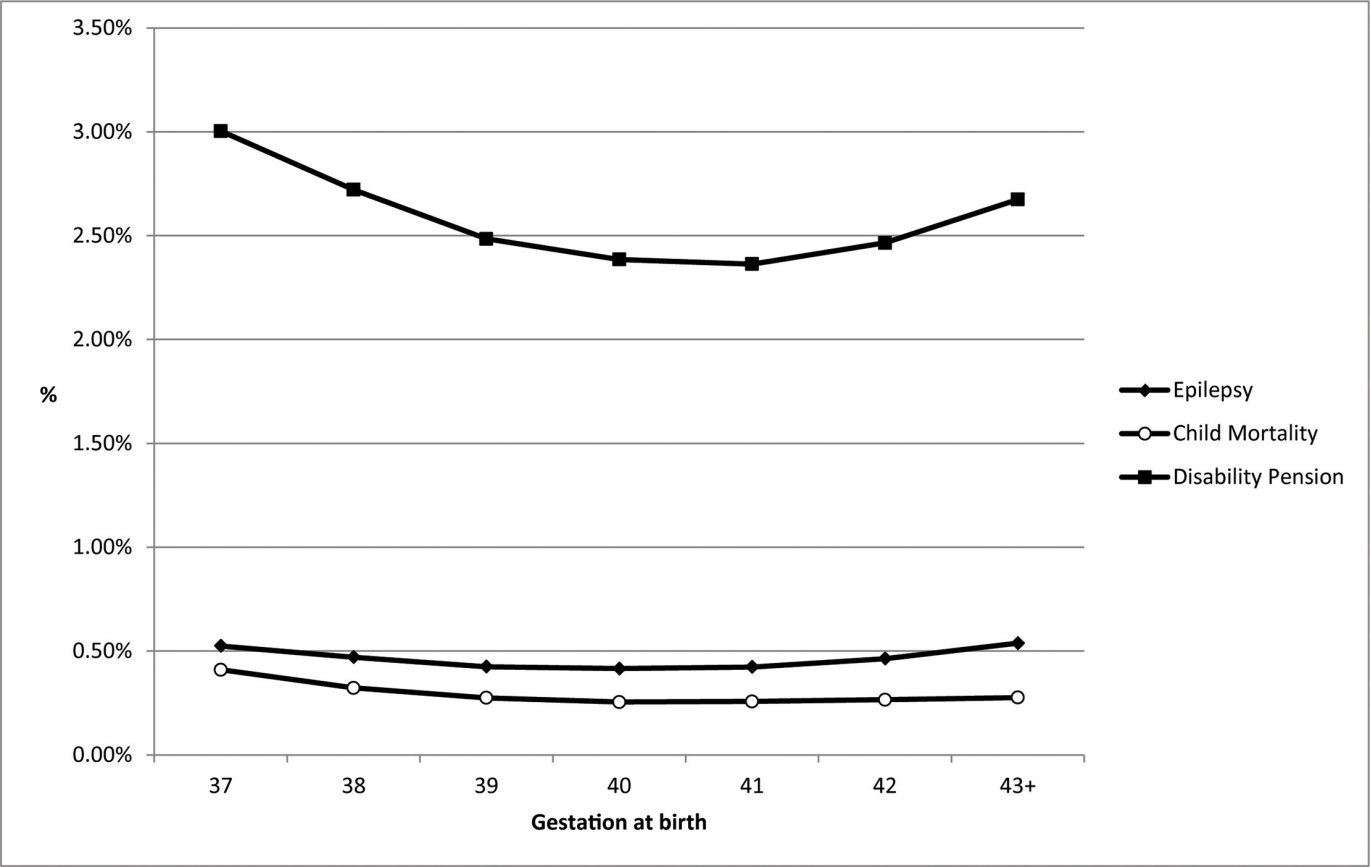
Proportion of children with epilepsy or disability pension before 20 years of age, and mortality before 5 years of age split by gestational age (n = 1,030,168).

In the univariable analysis there was statistical evidence that risk of epilepsy, childhood mortality, and rate of disability pension were associated with gestation at birth (Fig 1 and Table 2, all comparisons p<0·0001). The risk of epilepsy was lowest in infants born at 40 weeks gestation (0·39%), and highest in infants at younger (e.g. 0·62% in infants born at 37 weeks) and older (e.g. 0·53% in infants born at 42 weeks) gestations and a similar profile of risk was seen in childhood mortality and disability pension risk (S3 Table).

**Table 2:** Numbers and frequencies of outcomes by gestational age category at delivery (n = 1,030,168).

Outcomes	Completed gestational weeks at birth		
	37 or 38 weeks	39 or 40 weeks	41 or more weeks
n			
Epilepsy before 20 years of age	1088 (0.52%)	2281 (0.41%)	1225 (0.46%)
Childhood Mortality under 5 years of age	851 (0.41%)	1403 (0.25%)	708 (0.27%)
Disability pension before 20 years of age	6237 (3.0%)	13330 (2.4%)	6507 (2.5%)

Values are number (%) or mean (SD) as appropriate.· All comparisons p<0·0001

In the unadjusted model, early term infants had higher risks of epilepsy (p<0·0001), childhood death (p<0·0001) and disability pension (p<0·0001) than those born at 39–40 weeks’ gestation (Table 3), and this association persisted, albeit slighted attenuated, in the adjusted and fully adjusted analyses (epilepsy OR 1·19 (1·11–1·29), child death OR 1·42 (1·30–1·55), disability pension OR 1·20 (1·16–1·24)). In contrast, in the unadjusted results, late or post term infants had higher risks of epilepsy (p<0·0003) and disability pension (p = 0·0308) than infants born at 39–40 weeks’ gestation, but with insufficient evidence for an increase in the risk of child death (p = 0·1556). These associations persisted in the adjusted models with similar increased risks of epilepsy (OR 1·13 (1·06–1·22)) and disability pension (OR 1·05 (1·01–1·08)) to that seen in the unadjusted analysis, but there remained insufficient evidence for an increase in the risk of child death((p = 0.093). There was no evidence that the association between these gestational age groups and the risk of epilepsy was modified by low birth weight (p = 0·5083), multiple birth (p = 0·7208), maternal age (p = 0·7986), sex (p = 0·9301), caesarean section (p = 0·6744) or pre-eclampsia (p = 0·3096).

**Table 3:** Regression models for the association between early or late term birth, and the risk of epilepsy or disability pension before 20 years of age, and mortality before 5 years of age (n = 1,030,168).

Measure	Unadjusted		Adjusted*		Fully Adjusted**	
	OR (95% CI)	p	OR (95% CI)	p	OR (95% CI)	P
**Effect of birth at 37 or 38 weeks of gestation**						
Epilepsy	1·28 (1·19–1·38)	<0·0001	1·27 (1·18–1·37)	<0·0001	1·19 (1·11–1·29)	<0·0001
Child Death	1·63 (1·50–1·78)	<0·0001	1·62 (1·49–1·76)	<0·0001	1·42 (1·30–1·55)	<0·0001
Disability pension	1·27 (1·23–1·31)	<0·0001	1·24 (1·20–1·28)	<0·0001	1·20 (1·16–1·24)	<0·0001
**Effect of birth at 41 or more weeks of gestation**						
Epilepsy	1·14 (1·06–1·22)	0·0003	1·14 (1·06–1·22)	0.0002	1·13 (1·06–1·22)	0.0004
Child Death	1·07 (0·98–1·17)	0·1556	1·08 (0·99–1·19)	0·0800	1·08 (0·99–1·18)	0·0930
Disability pension	1·03 (1·00–1·07)	0·0308	1·05 (1·01–1·08)	0·0037	1·05 (1·01–1·08)	0·0053

Data are odds ratio (OR) (95% confidence interval)

* Adjusted for sex, primiparity, maternal age, education and socio-economic status.

** Adjusted for above, plus; multiple birth, maternal pre-eclampsia, caesarean birth, birth weight, maternal and infant infection.

There was however some evidence that the association differed depending on primiparity (p = 0·0560). Splitting the analysis by primiparity suggested that any effect of late or post term birth on epilepsy may be restricted to primiparous women (OR 1·20 (1·08–1·34) vs 1·11 (0·96–1·28)), whereas any increase in epilepsy on early term infants may be restricted to multiparous women (OR (1·12 (0·99–1·27) vs 1·24 (1·13 vs 1·36)).

### Sensitivity analyses

When using gestational age as an ordinal measure, there was evidence that for every week the infant was born after their due date the risk of epilepsy increased (fully adjusted OR 1.06 (1.03–1.10), p<0.001).

When repeating the analysis, splitting the late and post term infants into two separate groups, infants born late term remained at higher risk of epilepsy than full term infants, although the results did not, in isolation, reach conventional levels of statistical significance (OR 1.07 (0.99–1.16), p = 0.107). In contrast, those infants born post term continued to demonstrate clear evidence of an increased risk of epilepsy (OR 1.30 (1.17–1.45), p<0.001) when compared to full term infants.

Repeating the analysis using all infants recorded in the Medical Birth Register with available perinatal data resulted in compatible results to the main analysis (Early term; OR 1·09 (1·07–1·11), p<0·0001; Late or Post Term OR 1·03 (1·02–1·05), p = 0.0001), as did repeating it after exclusion of infants with known encephalopathy (Early term OR 1·20 (1·11–1·29), p<0·0001; late or post term OR 1·13 (1·06–1·22), p = 0·0005) and after excluding those infants born at 45 weeks gestation (Early term OR 1.19 (1.11–1.29), p<0.001; late or post term; OR 1.13 (1.06–1.22), p<0.001)). The relative risk of epilepsy in first ten years of life appeared to be compatible with that seen in the second decade, for infants born at 37/38 weeks gestation (First 10 years; OR 1.19 (1.06–1.33) vs Second 10 years; OR 1·20 (1·08–1·33)) and those born 41 of more weeks ((First 10 years; OR 1·14 (1·02–1·02) vs Second 10 years; OR 1·14 (1·03–1·25))) when compared to full term infants.

## Discussion

In this work, we have identified an increased risk of epilepsy in infants born either Early or Late/Post term, when compared to full term infants. This association appeared to increase the further from the due date the infant was born (either before or after) and persisted in a number of sensitivity analyses. The profile of association appears to be consistent with previous work in short term measures (e.g. encephalopathy) and suggests a ‘U’ shaped association with gestation around term; with the lowest risk being close to the infants’ due date and increasing for both infants born before and after this point. A sensitivity analysis was consistent with a gradient of increased risk as the gestational age increased beyond the expected date of delivery, and the association persisted after excluding infants in whom evidence of brain injury was evident after birth and after adjustment for potential confounders.

The limitations of this work include the use of routinely collected data, and the presence of missing data points. We defined epilepsy using the routine in-patient registry, although the underlying cause (if known) for the seizures is not recorded: hence some uncertainty for the biological mechanisms in play remains. We also did not have detailed information on the reasons for birth, or the clinical steps made by the clinicians to minimise risk to these pregnant women. However, the Swedish registries are considered robust sources of information and the above effects are likely, if anything, to minimise any effect size rather than introduce false positive findings. In addition, measurement of gestational age in this cohort may be less accurate than modern measures available to clinicians and may have changed gradually over the study period. However, any error around gestational age measurement is likely to reduce power to find an association (rather than introduce one) and a sensitivity analysis restricted to infants with gestations less than 45 completed weeks reported comparable results to the main analysis.

We hypothesised that rates of epilepsy, a common consequence of more obvious brain injury in the newborn period may be higher in infants born after 39 or 40 weeks gestation. Indeed, while other work has shown increased risk of perinatal asphyxia in early and post term infants[14,26,27], it seems possible that even mild levels of compromise may have measurable impacts on cognition, movement and social metrics when compared to their peers[28–30]. Recent work has suggested that the risk of epilepsy may also increase as a continuum after compromise at birth[22], and the associations in this work also appeared to persist after restriction to infants without obvious brain injury after birth.

The presumed causal pathways in play here are of great importance. The effects seen in early term infants are consistent with other work suggesting even small degrees of prematurity may have long term impacts[13]. These infants had higher risks of maternal disease (notably pre-eclampsia and infection), as well as high rate of operative delivery compared to full term infants. It would seem possible that some of the morbidity related to the infants is caused by underlying disease process which leads to the delivery, rather than a consequence of it *per se*. Consistent with this we did see some attenuation of the effect seen after adjustment for antenatal factors. However for these early term infants obvious intervention points are lacking and causal pathways are less clear.

The associations seen in the late and post term infants, by contrast showed little attenuation after adjustment, even after adjustment for intrapartum and neonatal factors (such as mode of birth). It would seem plausible that deterioration of the *in utero* environment, along with other consequences of late or post term birth (i.e. potentially a more difficult extraction) may contribute to worse birth condition and long term consequences. In contrast to early term births, enhanced surveillance, earlier induction or operative delivery may be options to minimize risk in women and infants who go past their due date. A recent randomized trial has suggested that induction as early as 39 weeks (compared to 40 weeks) is likely to reduce caesarean section rates[3], data consistent with the introduction of a clinical care bundle in the UK (Saving Babies’ Lives[31]). Importantly, neither study suggested impacts on neonatal morbidity although the current Cochrane review[32] reports on 30 randomised trials of induction of labour and concluded that further work was needed; particularly as no reviewed trial was able to report on neurodevelopmental outcomes. Given the apparent safety of induction of labour for the mother, this further highlights the results of this work; and it would seem prudent to discuss and/or offer induction of labour or at the least enhanced antenatal and intrapartum care for all women whose pregnancy continues past their 39th or 40th week of gestation. We also found little to suggest that the risks of epilepsy were modified by the clinical factors available to the clinicians (e.g. maternal age) with the exception of parity. This may simply be a false positive finding, and any mechanism is unclear in this work; hence should be interpreted cautiously. However increased stillbirth risks have been observed in primaparous post term pregnancies[33] and validation using different cohorts and methodologies should be performed. Previous work has suggested that preterm infants may demonstrate particularly high risk of developing epilepsy, when compared to term infants, during the first few years of life[34]. In this work we were unable to identify any change in the increased risk of epilepsy between the first two decades of life for either the Early Term or Late/Post term infants. This may reflect limited power to detect a real association, or that the casual pathways underlying the associations differ between the two studies.

### Conclusion

Infants born at early term (born at 37 or 38 weeks of completed gestation) appear to have increased risk of epilepsy, childhood death and needing disability support. The causal routes in this group are complex, and further work is needed to see if the impact appears to come from the cause of the early birth or the impact of it *per se*. In addition we found that infants born at 41 weeks gestation or more also appear to have increased risk of developing epilepsy as they grow, and higher risks of needing disability support. For this group more work is needed to identify if this association can be replicated, and if earlier birth may help prevent poor outcomes; but in the meantime, these data could provide useful information to help women and care givers make decisions with regard to the timing of induction of labour. It would also appear prudent to offer enhanced surveillance to these women in order to minimise any potential impact.

## Supporting information

S1 Fig Flow chart of participants. (DOCX)

S1 Table Characteristics of the study population according to missing data. Values are number (%) or mean (±SD) as appropriate.
(DOCX)

S2 Table Characteristics of the study population according to gestational age (n = 1,030,168). Values are number (%), mean (±SD) or geometric mean (95% confidence interval) as appropriate. All p values <0.001.
(DOCX)

S3 Table Numbers and frequencies of outcomes by gestational age at delivery (n = 1,030,168). Values are number (%) or mean (SD)s as appropriate. All comparisons p<0·0001.
(DOCX)

## References

[1] Kingdon C, Neilson J, Singleton V, Gyte G, Hart A, Gabbay M, et al Choice and birth method: mixed-method study of caesarean delivery for maternal request. BJOG. England; 2009;116: 886–895. 10.1111/j.1471-0528.2009.02119.x 19385961

[2] Davies-Tuck M, Wallace EM, Homer CSE. Why ARRIVE should not thrive in Australia. Women and birth: journal of the Australian College of Midwives. Netherlands; 2018 pp. 339–340. 10.1016/j.wombi.2018.08.168 30174207

[3] Grobman WA, Rice MM, Reddy UM, Tita ATN, Silver RM, Mallett G, et al Labor Induction versus Expectant Management in Low-Risk Nulliparous Women. N Engl J Med. United States; 2018;379: 513–523. 10.1056/NEJMoa1800566 30089070 PMC6186292

[4] Gülmezoglu A, Crowther C, Middleton P, Peatley E. Induction of labour for improving birth outcomes for women at or beyond term. Cochrane Database Syst Rev. 2012; 10.1002/14651858.CD004945.pub3 22696345 PMC4065650

[5] Odd DE, Doyle P, Gunnell D, Lewis G, Whitelaw A, Rasmussen F. Risk of low Apgar score and socioeconomic position: a study of Swedish male births. Acta Paediatr. 2008;97: 1275–1280. 10.1111/j.1651-2227.2008.00862.x 18489620 PMC2582400

[6] Odd D, Heep A, Luyt K, Draycott T. Hypoxic-Ischaemic Brain Injury: Delivery Before Intrapartum Events. J Neonatal Perinatal Med. 2017;10(4):347–353. 10.3233/NPM-16152 29286930

[7] Draycott T, Sibanda T, Owen L, Akande V, Winter C, Reading S, et al Does training in obstetric emergencies improve neonatal outcome? BJOG. 2006;113: 177–182. 10.1111/j.1471-0528.2006.00800.x 16411995

[8] Chiossi G. Timing of Delivery and Adverse Outcomes in Term Singleton Repeat Cesarean Deliveries. Obs Gynecol. 2013;121: 561–569. 10.1097/AOG.0b013e3182822193 PMC4066022 23635619

[9] Costeloe KL, Hennessy E., Haider S, Stacey F, Marlow N, Draper ES. Short term outcomes after extreme preterm birth in England: comparison of two birth cohorts in 1995 and 2006 (the EPICure studies). BMJ2. 2012;345. 10.1136/bmj.e7976 PMC3514472 23212881

[10] Moster D1, Wilcox AJ, Vollset SE, Markestad T LR. Cerebral palsy among term and postterm Births. JAMA. 2010;304: 976–82. 10.1001/jama.2010.1271 20810375 PMC3711561

[11] Linder N, Hiersch L, Fridman E, Klinger G, Lubin D, Kouadio F, et al Post-term pregnancy is an independent risk factor for neonatal morbidity even in low-risk singleton pregnancies. Arch Dis Child Fetal Neonatal Ed. 2015; 10.1136/archdischild-2015-308553 26645539

[12] Daskalakis GJ, Mesogitis SA, Papantoniou NE, Moulopoulos GG, Papapanagiotou AA, Antsaklis AJ. Misoprostol for second trimester pregnancy termination in women with prior caesarean section. BJOG. England; 2005;112: 97–99. 10.1111/j.1471-0528.2004.00285.x 15663405

[13] Wolke D, Samara M, Bracewell M, Marlow N, Group EPicS. Specific language difficulties and school achievement in children born at 25 weeks of gestation or less. J Pediatr. 2008;152: 256–262. 10.1016/j.jpeds.2007.06.043 18206699

[14] Chiossim G. Timing of Delivery and Adverse Outcomes in Term Singleton Repeat Cesarean Deliveries. Obs Gynecol. 2013;121. 10.1097/AOG.0b013e3182822193 PMC4066022 23635619

[15] Martinez-Biarge M, Madero R, González A, Quero A, García-Alix A. Perinatal morbidity and risk of hypoxic-ischemic encephalopathy associated with intrapartum sentinel events. Am J Obs Gynecol. 2012;206: 148.e1–7. 10.1016/j.ajog.2011.09.031 22079054

[16] Mishanina E, Rogozinska E, Thatthi T, Uddin-Khan R, Khan K, Meads C. Use of labour induction and risk of caesarean delivery: a systematic review and meta-analysis. Can Med Assoc J. 2014;189: 665–673. 10.1503/cmaj.130925 PMC4049989 24778358

[17] Smith G. Labour should be induced at term: FOR: The balance of risks versus benefits favours offering term induction to all women. BJOG. 2015;122: 982. 10.1111/1471-0528.13399 26011456

[18] Jacquemyn Y. Labour should be induced at term: AGAINST: No proof of benefit. BJOG. 2015;122: 982. 10.1111/1471-0528.13400 26011455

[19] National Institute for Health and Clinical Excellence (NICE). Inducing labour (CG70). Manchester: NICE; 2008.

[20] Royal College of Gynaecologists and Obstetricians. Each Baby Counts [Internet]. [cited 14 Jan 2017]. Available: https://www.rcog.org.uk/eachbabycounts

[21] Department of Health (UK). New ambition to halve rate of stillbirths and infant deaths [Internet]. [cited 12 Jan 2017]. Available: https://www.gov.uk/government/news/new-ambition-to-halve-rate-of-stillbirths-and-infant-deaths

[22] Liu X, Jary S, Cowan F, Thoresen M. Reduced infancy and childhood epilepsy following hypothermia-treated neonatal encephalopathy. Epilepsia. United States; 2017;58: 1902–1911. 10.1111/epi.13914 28961316

[23] Persson M, Razaz N, Tedroff K, Joseph KS, Cnattingius S. Five and 10 minute Apgar scores and risks of cerebral palsy and epilepsy: population based cohort study in Sweden. BMJ. England; 2018;360: k207 10.1136/bmj.k207 29437691 PMC5802319

[24] Validation of the content of the Swedish Medical Birth Register, 1974 Stockholm: National Board of Health and Walfare; 1977.

[25] Spong CY. Defining “term” pregnancy: recommendations from the Defining “Term” Pregnancy Workgroup. JAMA. United States; 2013;309: 2445–2446. 10.1001/jama.2013.6235 23645117

[26] Odd DE, Yau C, Winter C, Draycott T, Rasmussen F. Associations between birth at, or after, 41 weeks gestation and perinatal encephalopathy: a cohort study. BMJ Paediatr Open. 2018;2. 10.1136/bmjpo-2017-000010 PMC5842989 29637179

[27] Badawi N, Kurinczuk JJ, Keogh JM, Alessandri LM, O’Sullivan F, Burton PR, et al Antepartum risk factors for newborn encephalopathy: the Western Australian case-control study. BMJ. 1998;317: 1549–1553. 9836652 10.1136/bmj.317.7172.1549 PMC28732

[28] Odd DE, Lewis G, Whitelaw A, Gunnell D. Resuscitation at birth and cognition at 8 years of age: a cohort study. Lancet. 2009;373: 1615–22. 10.1016/S0140-6736(09)60244-0 19386357 PMC2688587

[29] Odd DE, Gunnell D, Whitelaw A, Lewis G. The association between birth condition and neuropsychological functioning and educational attainment at school age. A cohort study. Arch Dis Child. 2010; 10.1136/adc.2009.176065 PMC3015086 20705720

[30] Odd DE, Rasmussen F, Gunnell DJ, Lewis G, Whitelaw A. A Cohort Study of Low Apgar Scores and Cognitive Outcomes. Arch Dis Child Fetal Neonatal Ed. 2008;93: F115–20. 10.1136/adc.2007.123745 17916594 PMC5141261

[31] Widdows K, Roberts S, Camacho E, Heazell A. Evaluation of the implementation of the Saving Babies’ Lives Care Bundle in early adopter NHS Trusts in England. Manchester, UK; 2018.

[32] Middleton P, Shepherd E, Crowther CA. Induction of labour for improving birth outcomes for women at or beyond term. Cochrane database Syst Rev. England; 2018;5: CD004945 10.1002/14651858.CD004945.pub4 29741208 PMC6494436

[33] Hilder L, Sairam S, Thilaganathan B. Influence of parity on fetal mortality in prolonged pregnancy. Eur J Obstet Gynecol Reprod Biol. Ireland; 2007;132: 167–170. 10.1016/j.ejogrb.2006.07.010 16956710

[34] Sun Y, Vestergaard M, Pedersen CB, Christensen J, Basso O, Olsen J. Gestational age, birth weight, intrauterine growth, and the risk of epilepsy. *Am J Epidemiol* 2008; 167: 262–70. 10.1093/aje/kwm316 18042672 PMC2632964

