# Supplementary material for: Associations between early term and late/post term infants and development of epilepsy: A cohort study
Source: PLoS One. 2018 Dec 31;13(12):e0210181. doi: 10.1371/journal.pone.0210181 (PMC6312375; doi:10.1371/journal.pone.0210181)
Supplement: S1 Fig — (DOCX) [file pone.0210181.s001.docx]

**S1 Figure. Flow chart of participants**

Term infants born between 1973-2012 without congenital abnormalities (n=2,516,997)

Population-based cohort with epilepsy inpatient data for up to 20 years of follow-up in all participants (n=1,049,497)

Infants without linkage to hospital/epilepsy data up to the age of 20 years

(n=1,467,500)

Infants with missing data on *a priori* confounders (n=19,329)

Children in the main analyses

(n=1,030,168)
