# Supplementary material for: Associations between early term and late/post term infants and development of epilepsy: A cohort study
Source: PLoS One. 2018 Dec 31;13(12):e0210181. doi: 10.1371/journal.pone.0210181 (PMC6312375; doi:10.1371/journal.pone.0210181)
Supplement: S1 Table — Values are number (%) or mean (±SD) as appropriate. (DOCX) [file pone.0210181.s002.docx]

**S1 Table. Characteristics of the study population according to missing data**

| **Measure** | **Study cohort** | | **Infants with missing data** | | **p** |
| --- | --- | --- | --- | --- | --- |
| **Antenatal Factors** | n | Summary Measure | n | Summary Measure |  |
| Gestation at birth (weeks) | 1,030,168 | 39·6 (1·3) | 19,329 | 39·6 (1·4) | 0.1154 |
| Male | 1,030,168 | 527719 (51·2%) | 19,329 | 9922 (51·3%) | 0·7109 |
| Birthweight (g) | 1,030,168 | 3571 (494) | 17,407 | 3460 (482) | <0·0001 |
| Maternal pre-eclampsia | 1,030,168 | 17,031 (1·7%) | 19,329 | 287 (1·5%) | 0·0686 |
|  |  |  |  |  |  |
| **Intrapartum Factors** |  |  |  |  |  |
| Maternal Infection | 1,030,168 | 4587 (0·5%) | 19,329 | 62 (0·3%) | 0·0098 |
| Neonatal Infection | 1,030,168 | 2204 (0·2%) | 19,329 | 41 (0·2%) | 0.9563 |
| Caesarean Section | 1,030,168 | 98,265 (9·5%) | 19,329 | 1914 (9·9%) | 0·0884 |
|  |  |  |  |  |  |
| **Demographic factors** |  |  |  |  |  |
| Maternal Age (years) | 1,030,168 | 28·4 (4·5) | 19,329 | 27·5 (4·5) | <0·0001 |
| Primiparae | 1,030,168 | 413,503 (40·1%) | 19,329 | 11,355 (58·8%) | <0·0001 |
| Maternal Occupation | 1,030,168 |  | 6,931 |  | <0·0001 |
| Manual |  | 107,907 (10·5%) |  | 2228 (32·2%) |  |
| Non-manual |  | 326,166 (31·7%) |  | 2,282 (32·9%) |  |
| Other |  | 596,095 (57·9%) |  | 2,321 (33·5%) |  |
| Maternal Education Status | 1,030,168 |  | 12,794 |  | <0·0001 |
| <9 Years |  | 115,047 (11·2%) |  | 2952 (23·1%) |  |
| 9-10 Years |  | 527,483 (51·2%) |  | 5,178 (40·5%) |  |
| Full Secondary |  | 379,958 (36·9%) |  | 4,494 (35·1%) |  |
| Higher Education |  | 7,680 (0·8%) |  | 170 (1·3%) |  |
|  |  |  |  |  |  |
| **Birth Characteristics** |  |  |  |  |  |
| Apgar Score |  |  |  |  |  |
| 1 minute | 1,021,063 | 8·71 (8·70-8·71) | 18,831 | 8·69 (8·66-8·71) | 0.9725 |
| 5 minute | 1,007,976 | 9·74 (9·94-9·74) | 18,656 | 9·75 (9·74-9·77) | <0·0001 |
| Encephalopathy | 1,030,168 | 356 (0·03%) | 19,329 | 11 (0·06%) | 0·0996 |
|  |  |  |  |  |  |
| **Outcomes** |  |  |  |  |  |
| Epilepsy | 1,030,168 | 4595 (0·5%) | 19,329 | 73 (0·4%) | 0·1570 |
| Disability Pension | 1,030,168 | 26,074 (2·5%) | 19,329 | 364 (1·9%) | <0·0001 |
| Child Mortality | 1,030,168 | 2,962 (0·3%) | 19,329 | 70 (0·4%) | 0·0555 |

Values are number (%) or mean (±SD) as appropriate.
