# Supplementary material for: Associations between early term and late/post term infants and development of epilepsy: A cohort study
Source: PLoS One. 2018 Dec 31;13(12):e0210181. doi: 10.1371/journal.pone.0210181 (PMC6312375; doi:10.1371/journal.pone.0210181)
Supplement: S2 Table — Values are number (%), mean (±SD) or geometric mean (95% confidence interval) as appropriate. All p values <0.001. (DOCX) [file pone.0210181.s003.docx]

**S2 Table. Characteristics of the study population according to gestational age (n=1,030,168)**

| **Measure** | **Gestational Age** | | | | | | | | |
| --- | --- | --- | --- | --- | --- | --- | --- | --- | --- |
|  | **37** | **38** | **39** | **40** | **41** | **42** | **43** | **44** | **45** |
| **Antenatal Factors** |  |  |  |  |  |  |  |  |  |
| Male | 31,148 (52.5%) | 75,633 (51.0%) | 128,183 (50.2%) | 152,301 (50.6%) | 98,568 (52.1%) | 36,656 (54.6%) | 3,824 (54.6%) | 334 (53.4%) | 72 (47.7%) |
| Birthweight (g) | 3090 (457) | 3319 (447) | 3499 (440) | 3651 (449) | 3775 (462) | 3865 (478) | 3876 (497) | 3830 (502) | 3606 (585) |
| Pre-eclampsia | 1856 (3.1%) | 3126 (2.1%) | 4285 (1.7%) | 4277 (1.7%) | 2545 (1.4%) | 852 (1.3%) | 82 (1.2%) | 5 (0.8%) | 2 (2.0%) |
| **Intrapartum Factors** |  |  |  |  |  |  |  |  |  |
| Maternal Infection | 317 (0.53%) | 843 (0.57%) | 1111 (0.44%) | 1207 (0.40%) | 775 (0.41%) | 300 (0.45%) | 28 (0.40%) | 5 (0.80%) | 1 (0.66%) |
| Neonatal Infection | 281 (0.37%) | 253 (0.17%) | 429 (0.17%) | 599 (0.20%) | 457 (0.24%) | 215 (0.32%) | 28 (0.40%) | 3 (0.48%) | 2 (1.32%) |
| Caesarean Section | 12,438 (21.0%) | 33,400 (22.5%) | 18,883 (7.4%) | 14,077 (4.6%) | 11,305 (6.0%) | 6,581 (10.2%) | 1,193 (17.0%) | 104 (16.6%) | 14 (9.3%) |
| **Demographic factors** |  |  |  |  |  |  |  |  |  |
| Maternal Age (years) | 28.6 (4.7) | 28.6 (4.6) | 28.3 (4.5) | 28.4 (4.5) | 28.5 (4.5) | 28.5 (4.5) | 28.4 (4.6) | 27.8 (4.6) | 27.3 (4.2) |
| Primiparae | 23,506 (39.6%) | 55,213 (37.2%) | 98,595 (38.6%) | 120,144 (39.6%) | 80,280 (42.5%) | 31,547 (47.0%) | 3827 (54.6%) | 329 (52.6%) | 62 (41.1%) |
| Maternal Occupation |  |  |  |  |  |  |  |  |  |
| Manual | 6465 (10.9%) | 16,238 (11.0%) | 26,960 (10.6%) | 31,382 (10.4%) | 19,088 (10.1%) | 6918 (10.3%) | 754 (10.8%) | 79 (12.6%) | 22 (14.6%) |
| Non-manual | 19,975 (33.7%) | 48,546 (32.7%) | 82,545 (32.3%) | 94,677 (31.2%) | 57,604 (30.5%) | 20,419 (30.4%) | 2126 (30.4%) | 208 (33.3%) | 66 (43.7%) |
| Other | 32,873 (55.4%) | 83,536 (56.3%) | 145,800 (57.1%) | 177,171 (58.4%) | 112,361 (59.4%) | 39,827 (59.3%) | 4126 (58.9%) | 338 (54.1%) | 63 (41.7%) |
| Maternal Education |  |  |  |  |  |  |  |  |  |
| <9 Years | 7852 (13.2%) | 18,382 (12.4%) | 29,071 (11.4%) | 32,310 (10.7%) | 19,458 (10.3%) | 7093 (10.6%) | 775 (11.1%) | 82 (13.1%) | 24 (15.9%) |
| 9-10 Years | 31,122 (52.5%) | 76,582 (51.6%) | 131,672 (51.6%) | 155,067 (51.1%) | 95,188 (50.4%) | 33,921 (50.5%) | 3509 (50.1%) | 334 (53.4%) | 88 (58.3%) |
| Full Secondary | 19,929 (33.6%) | 52,340 (35.3%) | 92,737 (36.3%) | 113,501 (37.4%) | 72,897 (38.6%) | 25,642 (38.2%) | 2670 (38.1%) | 205 (32.8%) | 37 (24.5%) |
| Higher Education | 410 (0.70%) | 1016 (0.7%) | 1825 (0.71%) | 2353 (0.8%) | 1510 (0.80%) | 508 (0.8%) | 52 (0.7%) | 4 (0.6%) | 2 (1.3%) |
| **Birth Characteristics** |  |  |  |  |  |  |  |  |  |
| Apgar Score |  |  |  |  |  |  |  |  |  |
| 1 minute | 8.65 (8.64-8.67) | 8.77 (8.77-8.78) | 8.78 (8.78-8.79) | 8.73 (8.73-8.74) | 8.64 (8.63-8.64) | 8.48 (8.47-8.49) | 8.33 (8.28-8.38) | 8.42 (8.29-8.57) | 8.54 (8.22-8.87) |
| 5 minute | 9.67 (9.66-9.68) | 9.75 (9.75-9.76) | 9.78 (9.77-9.78) | 9.76 (9.75-9.76) | 9.72 (9.71-9.72) | 9.65 (9.64-9.66) | 9.58 (9.55-9.61) | 9.57 (9.45-9.68) | 9.73 (9.58-9.87) |
| Encephalopathy | 37 (0.06%) | 43 (0.03%) | 60 (0.02%) | 101 (0.03%) | 67 (0.04%) | 40 (0.06%) | 7 (0.10%) | 1 (0.16%) | 0 (0.00%) |

Values are number (%), mean (±SD) or geometric mean (95% confidence interval) as appropriate

All p values <0.001
