# Supplementary material for: Associations between early term and late/post term infants and development of epilepsy: A cohort study
Source: PLoS One. 2018 Dec 31;13(12):e0210181. doi: 10.1371/journal.pone.0210181 (PMC6312375; doi:10.1371/journal.pone.0210181)
Supplement: S3 Table — Values are number (%) or mean (SD)s as appropriate. All comparisons p<0·0001. (DOCX) [file pone.0210181.s004.docx]

**S3 Table. Numbers and frequencies of outcomes by gestational age at delivery (n=1,030,168)**

| **Outcomes** | **Completed gestational weeks at birth** | | | | | | | |
| --- | --- | --- | --- | --- | --- | --- | --- | --- |
|  | **37** | **38** | **39** | **40** | **41** | **42** | **43** | **44+** |
| **n** | **59,313** | **148,320** | **255,320** | **303,231** | **189,053** | **67,164** | **7006** | **776** |
| Epilepsy before 20 years of age | 369 (0·62%) | 719 (0·48%) | 1089 (0·43%) | 1193 (0·39%) | 822 (0·43%) | 353 (0·53%) | 45 (0·64%) | 5 (0.64%) |
| Childhood Mortality under  5 years of age | 315 (0·53%) | 536 (0·36%) | 642 (0·25%) | 761 (0·25%) | 501 (0·27%) | 176 (0·26%) | 21 (0·30%) | 10 (1.29%) |
| Disability pension before 20 years of age | 2003 (3·38%) | 4243 (2·85%) | 6360 (2·49%) | 6970 (2·30%) | 4503 (2·38%) | 1749 (2·60%) | 236 (3·37%) | 19 (2.45%) |

Values are number (%) or mean (SD) as appropriate·

All comparisons p<0·0001
